# Genetic analysis of human parainfluenza virus type 4 associated with severe acute respiratory infection in children in Luohe City, Henan Province, China, during 2017–2018

**DOI:** 10.1007/s00705-021-05154-3

**Published:** 2021-07-06

**Authors:** Shanshan Zhou, Naiying Mao, Yan Zhang, Aili Cui, Zhen Zhu, Ruiping Hu, Jin Xu, Wenbo Xu

**Affiliations:** 1 Inner Mongolia Laboratory of Molecular Biology, Inner Mongolia Medical University, Jinshan Avenue, Jinshan Development Zone, Hohhot, 010059 Inner Mongolia Autonomous Region China; 2 WHO WPRO Regional Reference Measles/Rubella Laboratory, NHC Key Laboratory of Medical Virology and Viral Diseases (National Institute for Viral Disease Control and Prevention, Chinese Centers for Disease Control and Prevention), 155# Changbai Road, Changping District, Beijing, 102200 China; 3 Henan Province Center for Disease Control and Prevention, 105# Nongye South Road, Zhengzhou, 450000 Henan China

## Abstract

During 2017–2018, nasopharyngeal aspirates (NPAs) from 627 hospitalized patients with severe acute respiratory infection at Luohe Center Hospital were tested by RT-PCR for human parainfluenza virus 4 (HPIV-4). Fourteen (2.2%) of the 627 samples were positive for HPIV-4. The complete HN gene was amplified from nine positive samples and sequenced. Sequence comparisons showed that the HPIV-4 strains circulating in the city of Luohe are closely related to HPIV-4A strains. Our study indicated that there were multiple lineages of HPIV-4 circulating in Henan Province in China during the study period. This will improve our understanding of the epidemiological and clinical characteristics of HPIV-4.

**Supplementary Information:**

The online version contains supplementary material available at 10.1007/s00705-021-05154-3.

Human parainfluenza viruses (HPIVs), which belong to the family *Paramyxoviridae*, are enveloped, negative, single-stranded RNA viruses [1, 2]. Based on genetic and antigenic variation, HPIVs have been divided into four serotypes in two different genera: *Respirovirus* (HPIV-1 and HPIV-3) and *Orthorubulavirus* (HPIV-2 and HPIV-4) [3, 4]. Globally, HPIVs account for a significant proportion of acute respiratory infections (ARIs) among children under the age of 5 years [5, 6]. HPIV-4 was first identified in 1959 by Johnson et al. [7] and was first associated with mild respiratory illness in young people. However, recent studies have indicated that it can cause more-severe infections, such as pneumonia and bronchiolitis, in children and elderly individuals [8–12], and in immunocompetent individuals as well as critically ill patients [13–15].

HPIV-4 is subdivided into two subtypes, HPIV-4A and HPIV-4B, based on hemagglutination inhibition and neutralization tests [16]. However, although the number of studies investigating HPIV-4 infection has increased globally, possibly due to improved diagnostic testing procedures, the molecular characteristics of regionally and globally circulating HPIV-4 strains have not been fully elucidated.

In this study, 627 nasopharyngeal aspirates (NPAs) were collected from hospitalized patients with severe acute respiratory infection (SARI) in the city of Luohe, Henan Province, China, during 2017-2018 and screened for HPIV-4 infection. Informed consent was obtained from patients or their guardians for the donation of the samples used in this study. This study was approved by the second session of the Ethics Review Committee of the National Institute for Viral Disease Control and Prevention (IVDC) of the China CDC under ethics approval no. IVDC2018 no. 012, and the study was conducted according to the appropriate guidelines. SARI cases were identified through the sentinel surveillance program for hospitalized SARI cases in China (http://www.gov.cn/zwgk/2011-02/11/content_1801649.htm).

The samples were transported to the IVDC of the China CDC under cold-chain conditions for further analysis. Specimens were stored in sterile minimal medium at -20 or -80°C pending molecular analysis. All procedures were performed in accordance with the relevant guidelines and regulations.

Viral RNA was extracted from NPA samples using a QIAamp Viral RNA Mini Kit (QIAGEN, Valencia, CA, USA), and HPIV-4-positive samples were screened by one-step real-time RT-PCR using a PrimeScript^TM^ RT-PCR Kit (Takara Biotechnology Dalian, China, catalog no. DRR064A) as described previously [17]. The complete hemagglutinin-neuraminidase (HN) gene (1740 nt) of HPIV-4 was amplified by nested RT-PCR for genotyping HPIV-4. The first round of RT-PCR was performed using the following in-house-designed primer pair: forward primer, 5’- ATAGGGGGGAACRCACTTCTCAGC-3’; reverse primer, 5’- GGCRGRTTGTTTRTYGAGGACC-3’. The nested PCR was carried out with the following inner primers: forward primer, 5’-AACAATCCAGARRGACRTCACATCAA-3’; reverse primer, 5’- TCTTTCAGTGGATGGTTGAGGA-3’. The RT-PCR products were purified for sequencing using a QIAquick Gel Extraction Kit (QIAGEN, Hilden, Germany), and the amplicons were sequenced on an ABI PRISM 3100 DNA Sequencer (PerkinElmer, Beijing, China). Nucleotide sequences were assembled and edited using Sequencher (Gene Codes Corporation, Ann Arbor, MI, USA). The sequences of HPIV-4 obtained in our study have been submitted to the GenBank database (accession numbers MT681670-MT681678).

Pairwise distances of nucleotide and deduced amino acid sequences were determined by alignment of with 43 HPIV-4 sequences from GenBank using the ClustalW algorithm implemented in MEGA software version 7.0. Phylogenetic analysis based on complete HN gene nucleotide sequences were performed in MEGA version 7.0 by the maximum-likelihood method using the Kimura 2-parameter substitution model, with statistical significance of phylogenies estimated by bootstrap analysis with 1,000 replicates.

From October 2017 to December 2018, a total of 627 NPAs from inpatients (391 males and 236 females) who met the SARI case definition were collected from Luohe Central Hospital in Henan Province. The hospitalized patients with SARI ranged in age from 0 to 91 years old, with children under 6 years old accounting for 78.0% (489/627). The median age was 7.9 years.

Of the 627 patients, 14 (2.2%, 95% CI 1.1-3.4) were positive for HPIV-4 by real-time RT-PCR. The cycle threshold (CT) values for the HPIV-4-positive samples ranged from 25 to 35. Most of the CT values of the positive samples ranged from 29 to 33. The ratio of males to females was 1.7:1. Among the 14 patients (median, 2.5 years) with determined HPIV-4, all of them were younger than 7 years old, and 50% were 0 to 3 years of age (Table 1). Among the pediatric patients, 10 (71.4%) were diagnosed with bronchopneumonia, three (21.4%) with bronchiolitis, and one (7.1%) with mucocutaneous lymph node syndrome (MCLS).

**Table 1:** Sex and age distribution of HPIV-4-positive patients

	Total number of specimens	Number positive for HPIV-4 (%)
Sex		
Male	391	8 (2.0%)
Female	236	6 (2.5%)
Age group (years)		
<1	77	1 (1.3%)
≥1 to <3	238	7 (2.9%)
≥3 to <6	174	5 (2.9%)
≥6 to <14	67	1 (1.5%)
≥14	71	0
Total	627	14 (2.2%)

The seasonality of HPIV-4 was observed throughout the study period. The positive rates of HPIV-4 in spring, summer, autumn, and winter were 1.2% (1/85), 17.5% (10/57), 0.5% (1/199), and 0.7% (2/286), respectively (Fig. 1). The highest detection rates of HPIV-4 were found in summer, and the detection rate of HPIV-4 varied significantly between seasons (χ ^2^ = 67.456, *P* = 0.000).

**Fig. 1:**
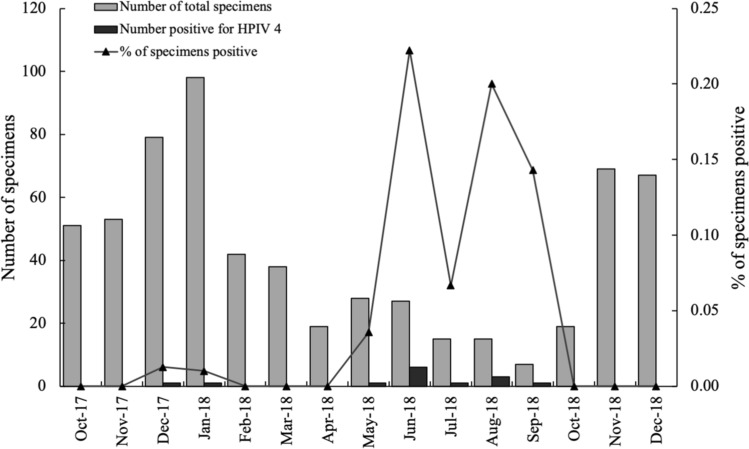
Monthly distribution of human parainfluenza virus 4 (HPIV-4) infections

The complete HN gene sequences of nine HPIV-4 isolates (CT values between 25 and 33) amplified from 14 HPIV-4-positive specimens were analyzed. The HN gene failed to be amplified from the remaining five HPIV-4-positive specimens, which had CT values ranging from 27 to 35 in the real-time RT-PCR. This could be due to mismatches in the primer-binding sites or low viral load.

All HPIV-4 HN gene sequences available in the GenBank database (43 sequences) were downloaded and aligned with the nine sequences obtained in this study. Among the nine HPIV-4 HN gene sequences, seven clustered with the HPIV-4A reference strains, and the remaining two clustered with the HPIV-4B reference strains (Fig. 2). By convention, the prototype of HPIV-4, which was isolated in Japan in 1959, formed a single lineage, designated as cluster I. The strains from Denmark in 2013, Japan in 2010, and Australia in 2008 constituted cluster II, with a mean nucleotide sequence divergence of 1.7%. Most of the viruses from Asia, with a mean nucleotide sequence divergence of 1.7%, were clustered together and designated as cluster III. The mean distance between the three clusters was 5.4%, which is larger than the mean distance within the three clusters (1.7%), in reference to a report that described phylogenetic analysis of HPIV-3 [18]. Cluster III strains were further grouped into four lineages (lineages 1, 2, 3 and 4) with 1.8-2.7% nucleotide sequence divergence. In our study, six HPIV-4A strains belonged to lineage 4, together with four previously reported strains from Japan. The remaining HPIV-4A strain was placed into lineage 3 with seven strains from Japan. In addition, two strains from this study were identified as HPIV-4B, which has rarely been reported in recent decades.

**Fig. 2:**
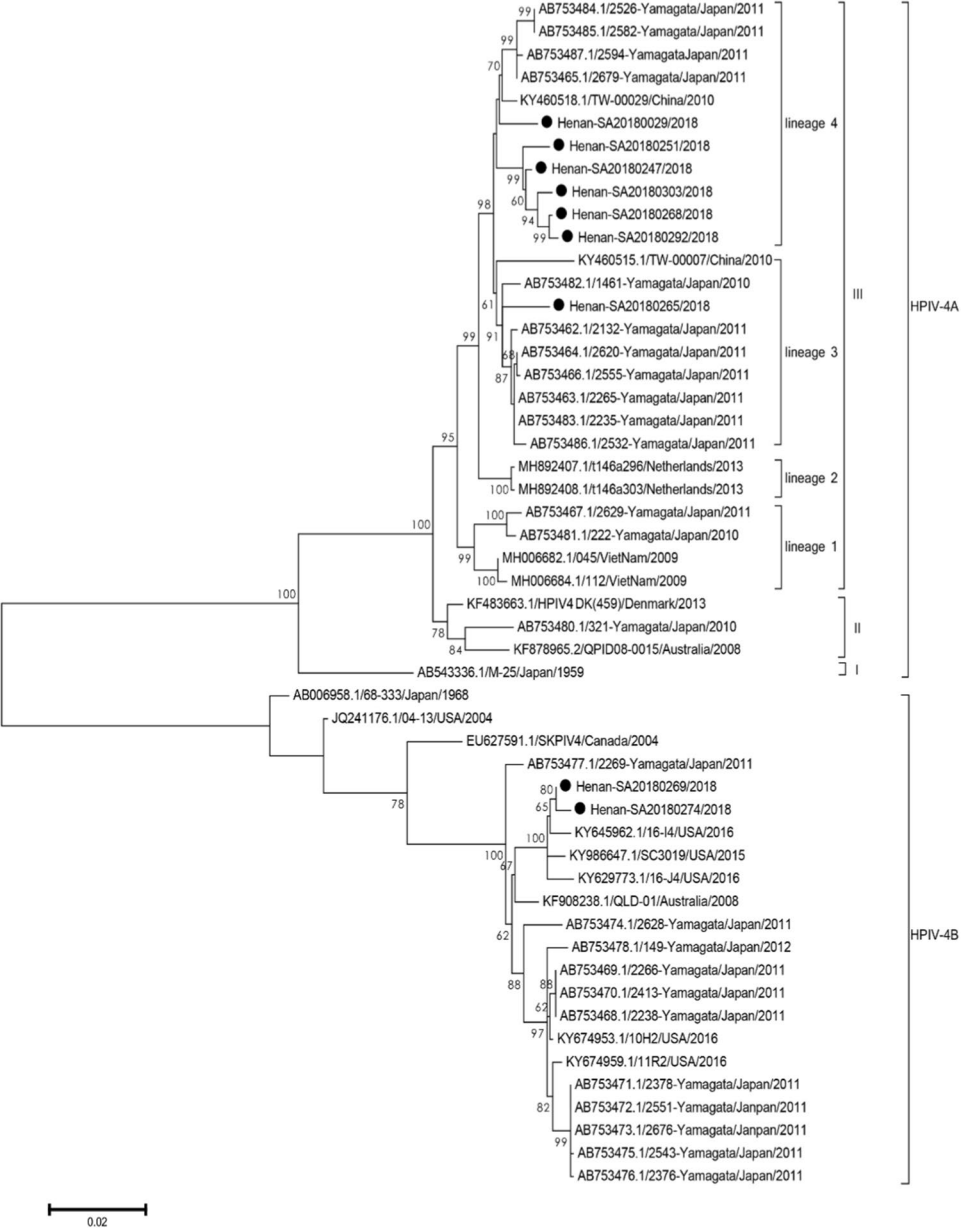
Phylogenetic tree based on complete HN gene nucleotide sequences of HPIV-4 strains

The divergence between the seven HPIV-4A strains from our study and the prototype strain M-25 was approximately 6.6%-6.9% and 10.1%-11.0% in the nucleotide and amino acid sequences, respectively. The two HPIV-4B isolates exhibited 4.9%-5.0% nucleotide sequence divergence and 8.0%-8.2% amino acid sequence divergence from the prototype strain. Notably, the length of the HN gene sequence (1740 nt) in our nine strains is longer than that of the prototype strain (1725 nt) by 15 nucleotides (5 amino acids) at the carboxy terminus. Compared with other strains, the divergence of nucleotides and amino acids among HPIV-4A was 1.0%-3.7% and 0.3-3.4%, respectively. Among the HPIV-4B isolates, it was 0.5-4.9% and 0.5-8.4%, respectively. Briefly, there are two common subtypes of HPIV-4 in China, HPIV-4A and HPIV-4B, of which HPIV-4A includes two lineages of cluster III.

In the present study, 14 samples that were positive for HPIV-4 from 627 (2.2%) SARI patients, collected between October 2017 and December 2018, were analyzed to determine the epidemiology of HPIV-4 in the city of Luohe, Henan Province, China, using q-RT-PCR. All patients with HPIV-4 infection were less than 7 years old, and 57% of them were younger than 3 years old, indicating that children in this age group are the main HPIV-4-susceptible population. This is in agreement with previous reports. The positive rate of HPIV-4 was 1.2% among 0.5-month- to 16-year-old patients, with the highest prevalence in 3- to 5-year-old children in Beijing, China. The median patient age in Colorado between 2009 and 2012 was 4.1 years old [19, 20]. Therefore, HPIV-4 may be gradually becoming an important cause of SARI in children, with higher severity than previously thought.

In our study, HPIV-4 infections occurred during summer and autumn, especially in summer, which is inconsistent with published studies from other countries. In Japan, Abiko et al. [8] described an outbreak of HPIV-4 infections during the 2011-2012 winter season. A similar result was reported in Canada during 2004-2005 [21]. This may be attributed to different geographical regions and study periods. However, as the number of positive samples in this study was limited, a large-scale investigation of the HPIV-4-positive rate is needed to understand the seasonal patterns of HPIV-4. In addition, considering that only strains from hospitalized patients were analyzed in this study, the possibility that other underestimated strains could be circulating in the general population cannot be ruled out.

In previous studies, HPIV-4 strains were detected at different times in different regions [21–23]. In our study, we characterized the HN gene from HPIV-4-positive samples from patients with SARI and further divided all of the HPIV-4A strains into three clusters. This is the first report describing the phylogeny of HPIV-4 based on complete HN sequences. Compared with the HPIV-4 prototype strain M-25, nine strains in our study, including other strains from GenBank, demonstrated significantly different characteristics. A five-amino-acid insertion in the HN protein of the Luohe strains may result in changes in the antigen-binding site and influence viral replication and transmission. This finding also indicates that the viruses are probably evolving.

Phylogenetic analysis revealed that five (Henan-SA20180251/247/303/268/292) of the seven HPIV-4A strains might share a chain of transmission. The remaining two HPIV-4A strains were similar to Japanese strains from 2010 and 2011. Phylogenetic analysis suggested that the domestic HPIV-4A strains belong to lineage 4 of cluster III, which seems to be prevalent. Moreover, two HPIV-4B strains were associated with three USA HPIV-4B strains isolated during 2015-2016. This reveals that the HPIV-4 strains were circulating globally and are more closely related to HPIV-4A than HPIV-4B, based on the HN gene sequences in our study. However, the genetic analysis of HPIV-4 in our study is only representative of Henan province, and not the entire country. In the future, additional sequences are needed to expand the dataset, and additional studies are required to determine the relative importance of HPIV-4A and HPIV-4B.

In conclusion, we have reported the HN gene sequences of nine HPIV-4 strains isolated from SARI patients. The divergence among HPIV-4 strains indicated that these viruses have circulated in the environment for many years and have undergone evolution. To better recognize its clinical importance and seasonal patterns, HPIV-4 should be included in the panels used for routine respiratory virus detection, although most clinical laboratories currently do not screen for HPIV-4 [23, 24]. This report provides valuable information about HPIV-4 isolates that might help to prevent HPIV-related respiratory diseases.

## Supplementary Information

Below is the link to the electronic supplementary material.

Supplementary file1 (TXT 43 kb)

## Data Availability

All data included in this study are available upon request from the corresponding author.
